# Annexin A2 (ANXA2) interacts with nonstructural protein 1 and promotes the replication of highly pathogenic H5N1 avian influenza virus

**DOI:** 10.1186/s12866-017-1097-0

**Published:** 2017-09-11

**Authors:** Yong Ma, Jiashan Sun, Linlin Gu, Hongmei Bao, Yuhui Zhao, Lin Shi, Wei Yao, Guobin Tian, Xiurong Wang, Hualan Chen

**Affiliations:** 1 State Avian Influenza Reference Laboratory, State Key Laboratory of Veterinary Biotechnology, Harbin Veterinary Research Institute, Chinese Academy of Agricultural Sciences, Harbin, 150001 China; 2 Animal Epidemic Diseases Control and Prevention Center of Liaoning Province, Shenyang, China

**Keywords:** H5N1 subtype HPAIV, NS1, ANXA2, Viral replication

## Abstract

**Background:**

Non-structural protein 1 (NS1) is a multifunctional protein and a crucial regulatory factor in the replication and pathogenesis of avian influenza virus (AIV). Studies have shown that NS1 can interact with a variety of host proteins to modulate the viral life cycle. We previously generated a monoclonal antibody against NS1 protein; In the current research study, using this antibody, we immunoprecipitated host proteins that interact with NS1 to better understand the roles played by NS1 in communications between virus and host.

**Results:**

Co-immunoprecipitation experiments identified annexin A2 (ANXA2) as a target molecule interacting with NS1. Results from confocal laser scanning microscopy indicated that NS1 co-localized with ANXA2 in the cell cytoplasm. Overexpression of ANXA2 significantly increased the titer of H5N1 subtype HPAIV, whereas siRNA-mediated knockdown of ANXA2 markedly inhibited the expression of viral proteins and reduced the progeny virus titer.

**Conclusions:**

Our results indicate that ANXA2 interacts with NS1 and ANXA2 expression increases HPAIV replication.

**Electronic supplementary material:**

The online version of this article (10.1186/s12866-017-1097-0) contains supplementary material, which is available to authorized users.

## Background

Avian influenza virus (AIV), which belongs to the *Orthomyxoviridae* family, contains a genome that includes eight separate negative-stranded RNA segments. These RNA segments encode at least 11 viral proteins [1]. AIV can be classified into two groups based on pathogenicity in bird: high pathogenicity and low pathogenicity groups.. Highly pathogenic H5N1 AIV replicates and circulates across a wide range of avian hosts and has significant economic impact on the poultry industry. Additionally, AIV poses a significant risk to human health because of the multiple mechanisms the virus uses to circumvent the diverse antiviral defenses in mammalian cells [2, 3]. Nonstructural 1 protein (NS1) of AIV is widely considered as an essential virulence factor with multiple functions during viral infection, including direct modulation of vital aspects of virus replication and antagonism of host immune responses at multiple levels [4, 5]. NS1 protein, which is encoded by viral segment number eight, is approximately 26 kDa and consists of 228–237 amino acids. According to structural analysis, NS1 contains two distinct functional domains: an N-terminal RNA-binding domain (RBD, amino acids 1–73) and a C-terminal effector domain (ED, amino acids 74–230). The C-terminal domain mainly interacts with host proteins to modulate the viral infection process by inhibiting the host immune response. For example, interaction between NS1 and the ubiquitin ligase TRIM25 allows the virus to evade recognition by the host viral-RNA sensor RIG-I or human guanylate-binding protein 1 to avoid antiviral activity [6–10]. In addition to inhibiting host immune responses, NS1 has also recently been suggested to play an important role in promoting efficient virus replication and virulence during infection. For example, NS1 can recruit eIF4GI to the 5’UTRs of viral mRNAs, causing the selective translation of viral mRNAs over cellular mRNAs and thereby increasing viral protein expression [11, 12]. In general, the multifunctional NS1 protein has a wide variety of regulatory functions and interacts with a multitude of proteins.

To identify novel host factors involved in H5N1 AIV infection, we developed a proteomics strategy to screen for cellular proteins that interact with NS1 by utilizing an anti-NS1 monoclonal antibody (D7) previously generated by our group [13]. We identified an interaction between NS1 and ANXA2 through mass spectrometry (linear ion trap Fourier transform ion cyclotron resonance-mass spectrometry [LTQ-MS]) analysis. Further confirmation of the interaction was achieved through a series of cellular and molecular assays. Our results show that ANXA2 is a pro-viral host factor contributing to influenza virus replication in vitro. Our study reveals that ANXA2 plays an important role in accelerating the replication of the highly pathogenic influenza strain H5N1 and this finding broadens our understanding of the function of ANXA2 in influenza virus replication. .

## Results

### ANXA2 is a novel binding partner of AIV NS1 protein

**Fig. 1:**
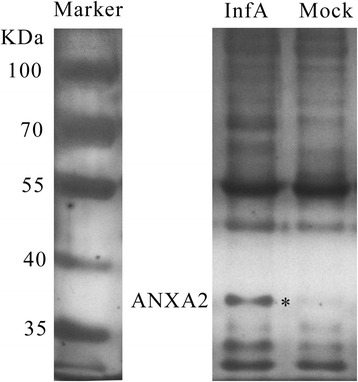
ANXA2 was confirmed as a novel host protein that binds to NS1. NS1-associated proteins from infected (InfA) or uninfected (Mock) A549 cell lysates were immunoprecipitated using the anti-NS1 D7 antibody, separated by SDS-PAGE (8%), and visualized by silver staining. The InfA group-specific band (indicated by an asterisk) was identified as ANXA2 by LTQ-MS

We used the anti-NS1 monoclonal antibody D7, which specifically recognizes the peptide^29^DAPF^32^ in the AIV NS1 protein, to immunoprecipitate NS1-associated proteins from infected A549 cell lysates. The NS1 protein used for the immunoprecipitation (IP) was derived from the A/duck/Guangdong/S1322/2010 (GD1322) H5N1 strain. Comparing the protein band patterns between infected and uninfected lysates, we found that a protein of approximately 35 kDa was present only in the infected cell lysate (Fig. 1). Further analysis with LTQ-MS indicated that the best match for this protein was annexin A2 (Table 1).

**Table 1:** Identification of ANXA2 protein bands

Technique	GenInfo identification	Mass (KDa)	PI	CoverPercent^b^ (%)	UniquePep^c^ Count
LTQ^a^	P07355	38.604	7.57	60.18%	18

^a^Database searching from uniprot

^b^Percentage of total protein sequence covered by matched peptides

^c^Mumbers of unique matched peptides

### NS1 interacts with ANXA2

**Fig. 2:**
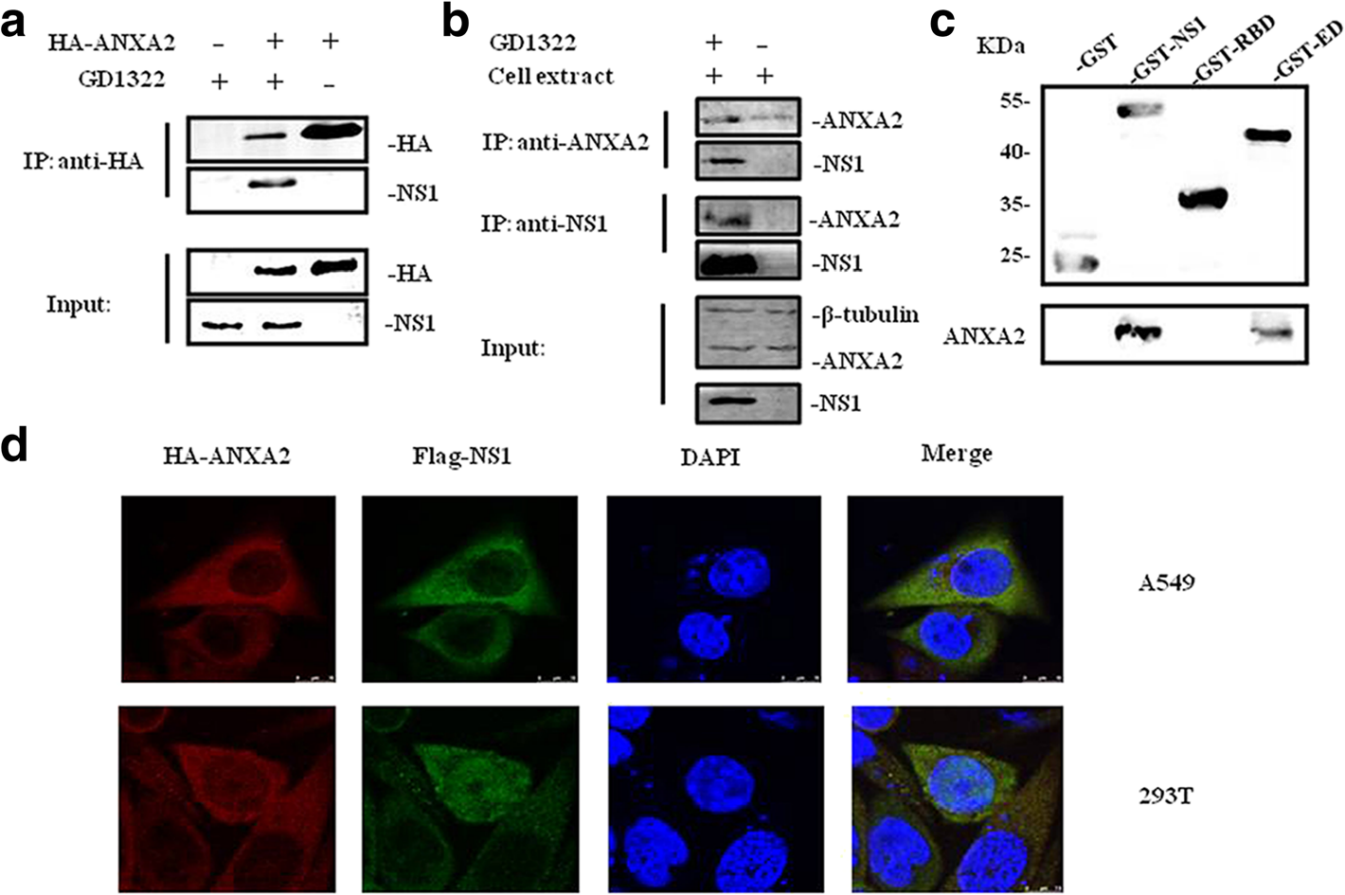
Validation of the interaction between ANXA2 and NS1. **a** Co-IP assay confirming the interaction between NS1 and ANXA2. A549 cells were transfected with HA-ANXA2 plasmid and infected with GD1322 24 h later. Cell lysates, harvested 36–48 h after infection, were subjected to IP and western blotting with anti-HA or anti-NS1 D7 antibodies. **b** Co-IP assay detecting the association between NS1 and endogenous ANXA2. A549 cells were infected with GD1322 and collected after 12 h. Next, cell lysates from infected or uninfected A549 cells were immunoprecipitated with an anti-ANXA2 rabbit antibody or the anti-NS1 D7 mouse antibody. After incubation with protein A/G-agarose beads, the Immunoprecipitation pellets were immunoblotted with the anti-NS1 D7 mouse antibody or the anti-ANXA2 rabbit antibody. **c** Pull down assay to verify that the ED of NS1 binds with ANXA2. Full-length NS1 and each functional domain were fused with GST and then incubated with uninfected A549 lysate. GST and ANXA2 were detected by western blotting. **d** Colocalization of ANXA2 and NS1 in A549 cells. HA-ANXA2 and FLAG-NS1 plasmids were co-transfected into A549 or 293 T cells. After incubation for 24 h, the cells were double-immunostained for FLAG-NS1 (green) and HA-ANXA2 (red). Nuclei were counterstained with 4′,6-diamidino-2-phenylindole (DAPI) (blue)

We employed coimmunoprecipitation (co-IP) to investigate the interaction between NS1 and ANXA2. As shown in Fig. 2a, NS1 protein was only detected in complexes immunoprecipitated using the anti-HA antibody. It has been suggested that ANXA2 interacts with NS1 protein. To examine the association between NS1 and endogenous ANXA2, virus-infected A549 cell lysate was immunoprecipitated with the anti-NS1 D7 mouse MAb, and the pellet was analyzed with an anti-ANXA2 rabbit PAb. As shown in the anti-NS1 panels in Fig. 2b, endogenous ANXA2 was co-immunoprecipitated with the anti-NS1 D7 MAb. A reverse IP assay was executed using the anti-ANXA2 rabbit PAb, and the complexes formed were detected with the anti-NS1 D7 mouse MAb. As expected, the NS1-specific band was only detected in the virus-infected cell lysate (Fig. 2b, anti-ANXA2 panels). In order to determine which domain of NS1 interacts with ANXA2, a pull down assay was used. First, A549 cell lysate was precipitated with GST-tagged fusion proteins, which have been purified by affinity chromatography. The results showed that only full-length NS1 (GST-NS1) and the ED (GST-ED) interacted with ANXA2, indicating that the ED is responsible for binding with ANXA2 (Fig. 2c).

A confocal laser scanning microscopy assay showed that ANXA2 and NS1 co-localized in the cytoplasm of transfected cells. As shown in Fig. 2d (upper panel), HA-ANXA2 evenly localized in the cytoplasm of A549 cells, whereas Flag-NS1 was distributed predominantly in the cytoplasm but slightly in the nucleus. As expected, NS1 and ANXA2 co-localized in the cytoplasm. The same colocalization results were seen with HEK 293 T cells, although Flag-NS1 was distributed in the cytoplasm and nucleus simultaneously. (Fig. 2d, lower panel). Taken together, our results indicate that NS1 interacts with ANXA2 in cytoplasm and that the ED is the functional domain in this pairing.

### ANXA2 significantly influences AIV replication in host cells

**Fig. 3:**
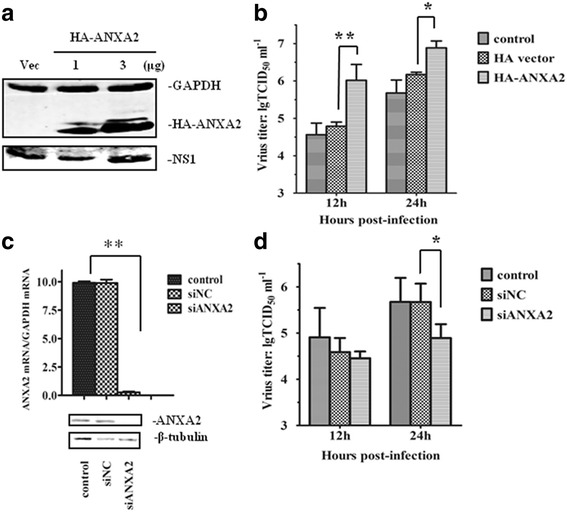
ANXA2 influences AIV replication in A549 cells. **a** Overexpression of ANXA2 in A549 cells. A549 cells were transfected with HA-ANXA2 plasmid or an empty vector (Vec) and then infected with GD1322 (MOI = 0.1). After 24 h, HA-ANXA2 and NS1 proteins were detected by western blotting. **b** Progeny virus titers increased significantly in A549 cells overexpressing ANXA2. Viral supernatants were collected at 12 h and 24 h after infection. Viral titers were assayed based on hemagglutination, and the results are expressed as TCID_50_ per mL. **c** Knockdown efficiency of ANXA2. A549 cells were transfected with siNC or siANXA2. The results shown are from qPCR and western blotting analyses performed 24 h after infection. **d** Progeny virus titers decreased significantly after transfection with siANXA2 at 24 hpi. The results are presented as the mean and SD from three independent experiments. *, p<0.05 compared with cells transfected with empty vector or siNC. **, p<0.01 compared with cells transfected with empty vector or siNC

A549 cells were transfected with HA-ANXA2 to overexpress ANXA2 protein and infected with GD1322 24 h later. As shown in Fig. 3a, ANXA2 expression increased according to transfection dose (from 1 μg to 3 μg), as did NS1 expression. Furthermore, the 50% tissue culture infectious dose (TCID_50_) indicated that ANXA2 exerted a pro-viral influence on AIV replication in A549 cells. As shown in Fig. 3b, overexpression of ANXA2 resulted in a significant increase in the titer of progeny viruses compared with the empty vector and control group after 12 and 24 h of infection. Additionally, siANXA2 and siNC were used to transfect A549 cells, and the resultant downregulation of ANXA2 was compared. As shown in Fig. 3c, ANXA2 expression was dramatically decreased by siANXA2. To investigate whether ANXA2 knockdown affected AIV replication, we calculated the titers of progeny viruses in transfected and control A549 cells. The results showed a significant reduction in the progeny viral titer in the supernatant of the ANXA2-knockdown cells at 24 h post infection (pi) (Fig. 3d).

**Fig. 4:**
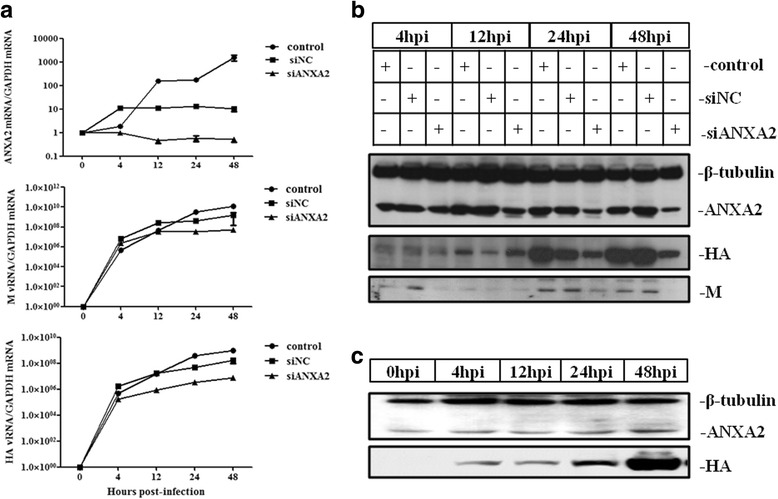
ANXA2 influences the replication of H5 subtype AIV. A549 cells were infected with GD1322 (MOI = 0.1) after transfection with siNC or siANXA2 for 24 h. The cells were then collected at 4, 12, 24 and 48 hpi to extract RNA or protein (**a**, **b**). A549 cells were also collected at 0, 4, 12, 24, and 48 hpi to extract protein (**c**). **a** qRT-PCR detection of gene synthesis of viral genes after transfection with siRNA. Total RNA was extracted from A549 cells, and ANXA2 mRNA, M vRNA and HA vRNA were used for relative quantification. GAPDH was used as a control. **b** Western blotting detection of viral protein expression after transfection of siRNA. After extracting total proteins from A549 cells, viral protein expression was determined using H5-specific antiserum from our lab. ANXA2 and tubulin were detected using commercial MAbs. The results are presented as the mean and SD from three independent experiments. **c** Western blotting detection of ANXA2 and HA expression after infection with H5N1 AIV. The same antibodies described above were used

We next measured AIV replication after silencing ANXA2. Gene expression was analyzed using quantitative real-time RT-PCR (qRT-PCR). As shown in Fig. 4a, ANXA2 expression significantly declined after transfection of siANXA2. Examination of viral hemagglutinin (HA) and matrix (M) expression showed an apparent decrease at 12 h after infection. We also utilized western blotting to compare the expression of viral proteins in ANXA2-knockdown and control cells. As ANXA2 expression decreased, there was a visible decline in the expression of viral proteins, especially HA and M1, at 24 h and 48 h pi (Fig. 4b). Furthermore, ANXA2 expression in the control group increased slightly after infection (Fig. 4c). Taken together, these results suggest that ANXA2 has a major influence on H5 subtype AIV replication in A549 cells and may play an important role in the viral life cycle.

## Discussion

Interactions between virions and host cellular proteins are common during the infection process. Identification of new host factors that bind to viral proteins is vital not only to understanding of viral infection and pathogenesis mechanisms, but also to developing effective anti-viral strategies. In this study, we showed that the NS1 protein of the H5N1 AIV strain could interact with human ANXA2 in vitro. The interaction is confirmed by co-IP. Results from confocal microscopy analysis further reveal that NS1 and ANXA2 co-localize in the cell cytoplasm. This finding differs from some previous studies, in which the NS1 protein was detected abundantly in the nucleus [14, 15]. We revalidated the distribution of NS1 of GD1322 by directly infecting or transfecting Flag-NS1 into A549 cells at different times, and found that by either infection or transfection, NS1 protein of GD1322 mainly distributed in cytoplasm of A549 cells, especially at late stage (Additional file 1: Figure S1). Researchers have demonstrated that the localization of NS1 is influenced by two factors. First, NS1 protein of different virus subtypes may have different distribution characteristics [16]. For example, Keiner [17] has reported that nuclear export signal of NS1 protein is responsible for its cytoplasmic accumulation. Second, the distribution of NS1 may be varied at different cells. For example, Volmer [18] has reported recently that one specific NS1 protein has different localization patterns in mammalian and avian cells.

Next, we explored the influence of ANXA2 on AIV replication. Interestingly, we found that the level of ANXA2 expression would markedly influence the progeny virus titer. In particular, silencing ANXA2 with siRNA led to a significant decrease in the expression of viral proteins, especially HA and M1. We also found that the expression of ANXA2 is slightly increased after infection, especially at late stage. We think it is a good example of AIV modulating the translation of host proteins selectively, although the mechanism is still unknown. Because of ANXA2’s positive function, we speculate that AIVs maintain and promote the expression of ANXA2 to accelerate viral replication, especially at the late stage when cells have been fully controlled by virus.

ANXA2 (also called calpectin 1or annexin II) is one of the most abundant proteins in human cells [19] and has been implicated in the regulation of several key biological processes, including cellular transport, endosome formation, membrane trafficking, vesicle aggregation, and exocytosis [20–23]. ANXA2 also plays a variety of roles in the viral life cycle. For example, ANXA2 takes critical part in the processes related to cell entry and assembly of human papillomavirus type 16 [24, 25] or avian leukosis virus subgroup J [26], and in the regulation of replication, assembly and release of porcine reproductive and respiratory syndrome virus [27], classical swine fever virus [28], hepatitis C virus [29] and bluetongue virus [30]. ANXA2 can also initiate replication of influenza virus [31, 32]. Previous research showed that ANXA2 had the ability to support replication of H3N2 influenza A virus strains. It is already known that, after entry into cells, membrane fusion between virions and endosomes occurs only when HA is cleaved. It has also been demonstrated that ANXA2 can facilitate the conversion of plasminogen (PLG) into plasmin, providing the protease activity necessary for the cleavage of precursor HA molecules to activate infection. However, in our study, we examined the highly pathogenic H5N1 AIV strain. The HA protein from this strain contains several basic amino acids (R-X-R/K-R) that can be cleaved by intracellular subtilisin-type proteases [33]. Furthermore, the TCID_50_ for the H5N1 strain did not change regardless of the use of TPCK trypsin (data not shown), suggesting that this strain can infect cells without trypsinization [34] and demonstrating that conversion of PLG into plasmin is not necessary for infection with highly pathogenic influenza virus. Additionally, the slight differences in the viral expression at early stages between the siRNA treatment groups and control groups also suggested that ANXA2 promoted viral replication in ways other than activating infection.

The present study showed that the progeny virus titer was significantly decreased in A549 cells in which ANXA2 had been transiently knocked down by siRNA. By contrast, the titer substantially increased when ANXA2 was transiently overexpressed. Previous studies have demonstrated that modulating the expression of ANXA2 influences cell proliferation and apoptosis: overexpression of ANXA2 affects cell proliferation [35], whereas silencing it both suppresses cell proliferation and upregulates the enzyme activity of caspase-3, caspase-8 and caspase-9 to enhance cell apoptosis [36]. ANXA2 also plays a role in regulating p53 via the JNK/c-Jun pathway, and knockdown of the protein increases the expression of p53 and its downstream genes to initiate apoptosis [37, 38]. Notably, apoptosis is a well-known host defense mechanism against viral infection [39], and NS1 helps regulate the apoptosis of infected cells, although it appears to have a contradictory role because both pro- and anti-apoptotic functions have been described [40, 41]. Wang reported that exogenously expressed NS1 associates with p53 to inhibit cell apoptosis, further studies have found a p53 inhibitory domain on NS1 located between amino acids 144 and 188 [42]. However, this domain required additional partners to cooperatively exert this inhibitory function [43]. Based on the interaction between ANXA2 and NS1 and the potential antagonism of p53, we hypothesize that NS1 acts as a bridging molecule to bring together p53 and ANXA2 to inhibit cell apoptosis.

## Conclusions

Our results confirm that an interaction occurs between NS1 and ANXA2 after infection of A549 cells with the H5N1 AIV strain. This study is the first to show that ANXA2 plays a role in promoting the replication of H5N1 AIV strains through a unique mechanism that does not require the conversion of PLG into plasmin to initiate infection.

## Methods

### Cells and viruses

Human lung epithelial cell line A549 cells were cultured in F-12 K Nutrient Mixture with Kaighn’s Modification (GIBCO, Grand Island, NY, USA). Madin-Darby canine kidney cell line MDCK cells and human embryonic kidney cell line HEK293 T cells were maintained in Dulbecco’s modified Eagle medium (DMEM) (GIBCO). All cells were provided by the National Animal Influenza Reference Laboratory, and cultivated in medium supplemented with 10% fetal bovine serum (GIBCO) plus 100 units/mL penicillin and 100 μg/mL streptomycin sulfate at 37 °C under 5% CO_2_. The isolate strain A/duck/Guangdong/S1322/2010 (GD1322; H5N1 subtype; high pathogenic) influenza virus was propagated in 9-day-old embryonated specific-pathogen-free chicken eggs at 37 °C and collected no more than three days later or when they died. All infectious operation about high pathogenic influenza viruses were implemented in BSL-3 laboratory.

### IP and LTQ-MS analysis

A549 cells were infected with AIV GD1322 strain at a multiplicity of infection (MOI) 1 at 37 °C for 1 h, and uninfected A549 cells were used as a negative control. We collected 10 h post-infection or uninfection (mock) cells, and lysed them by ultrasonication on ice until the supernatant was transparent and centrifuged the lysate at 8000×g for 15 min at 4 °C. The supernatant was used for subsequent binding analyses. For screening of NS1-binding host factors, a former identified anti-NS1 antibody D7 was generated in our laboratory. For IP of the NS1-complex, the supernatant was incubated with 5 μg of anti-NS1 D7 and 20 μL of protein A/G PLUS-Agarose beads (Santa Cruz Biotechnology, Dallas, TX, USA) overnight at 4 °C simultaneously. After washing three times with 1 mL of cold PBS gently but extensively, the beads were resuspended with cold PBS and resolved by SDS-PAGE, the separated proteins were detected by silver staining. We verified the results of the protein binding tests by three repeats. Through comparison of the infected and uninfected samples, protein bands specific to the infected group were identified and analyzed by LTQ-MS. The LTQ-MS analysis was executed by Shanghai Applied Protein Technology Co. Ltd., China.

### Plasmids construction

Expression vectors containing a HA or Flag tag were constructed using a pCAGGS vector. A Kozak consensus sequence (CCACC) to optimize expression of the fusion proteins was constructed in all of the plasmids in this study. The full-length NS1 gene from GD1322 (H5N1), isolated by RT-PCR of the viral total RNA extracted by QIAamp Viral RNA Mini kit (Qiagen, Hilden, Germany), was cloned into a pCAGGS-Flag vector and named as pCAGGS-Flag-NS1 (Flag-NS1). The human ANXA2 gene was amplified by RT-PCR from A549 cells and then cloned into a pCAGGS-HA vector, and the recombinant plasmid obtained was named pCAGGS-HA-ANXA2 (HA-ANXA2). We also cloned the sequences of NS1, RBD and ED regions into pGEX-6p-1 vector to express GST-fused proteins. The DNA sequences of the genes inserted into the plasmids were verified by sequence analysis. All the primers used in the construction are listed in Table 2.

**Table 2:** Primers used in this experiment

Primer	Sequence (5′-3′)
FLAG-NS1-F	CAGAATTCTAATGGATTCCAACACTGTG (EcoRI)
FLAG-NS1-R	GGGGTACCCACTTCTGACTCAATTGTTC (KpnI)
HA-ANXA2-F	GGGGTACCCCGTCTACTGTTCACGAAATCCTG (KpnI)
HA-ANXA2-R	GAAGATCTTCGTCATCTCCACCACACAGGTA (BglII)
ANXA2-159F	TGGATGAGGTCACCATTGTCA
ANXA2-159R	TCAATAGGCCCAAAATCACC
GAPDH-F	GGCATCCTGGGCTACACTGA
GAPDH-R	TGTTGCTGTAGCCAAATTCGTT

### Co-IP and pull down assay

293T cells were transfected with the HA-ANXA2 plasmid using Lipofectamine 2000 (Invitrogen, Carlsbad, CA, USA) according to the manufacturer’s protocols, and 24 h post-transfection they were infected with the GD1322 virus and then collected at 10 h pi. The cells were lysed in 200 μL of cell lysis buffer (20 mM Tris-HCl, pH 7.5, 150 mM NaCl, 1% Triton X-100, sodium pyrophosphate, β-glycerophosphate, EDTA, Na_3_VO_4_, and leupeptin) containing 1 mM phenylmethylsulfonyl fluoride (PMSF, Beyotime, Shanghai, China) for 30 min at 4 °C before IP and western blotting. The procedure of co-IP was described previously. Proteins were boiled in SDS sample buffer, separated by SDS-PAGE, and then detected by western blotting using the anti-HA-tagged or anti-Flag-tagged antibody.

To investigate interactions between NS1 and endogenous host proteins in GD1322-infected cells, A549 cells were infected with GD1322 (MOI = 0.1) for 12 h, with uninfected A549 cells used as a control. The cells were incubated with agarose beads and proteins binding to them were detected by western blotting, as described above.

### Confocal laser scanning microscopy assays

A549 cells were grown in glass-bottomed cell culture dishes (Nest Biotechnology, Wuxi, China) and transfected with Flag-NS1 and HA-ANXA2 when cells were 40–50% confluent. Then operated as described before [44], and then examined using a Leica SP2 confocal system (Leica Microsystems GmbH, Wetzlar, Germany). HEK 293 T cells were also used as a parallel control to detect the locations of Flag-NS1 and HA-ANXA2, the operated is the same as before.

### Western blotting analysis

After estimating the protein concentration with bicinchoninic acid protein assay reagent (BCA, Beyotime), 25–50 μg (per lane) of the cell lysates or Co-IP products were subjected to SDS-PAGE and then blotted onto a polyvinylidene difluoride membrane (Millipore). Membranes were incubated as previously described [45]. The samples were reacted using an Odyssey imaging system (Li-Cor Biosciences, Lincoln, NE, USA.).

### RNA interference

All the siRNAs used in this study were designed and synthesized by Shanghai GenePharma (Shanghai, China). A549 cells, at a confluence of 80% in 6-well plates, were transfected with 320 nM of effective siRNA specific for human ANXA2 gene (GenBank NM_004039.2; siANXA2, sense 5′ CCUCCAGAAAGUAUUUGAUTT 3′). The negative control siRNA was a scrambled siRNA for ANXA2 (siNC, sense 5′ GUGAACGAACUCCUUAAUUTT 3′). All siRNAs were transfected into cells using Lipofectamine RNAiMAX transfection reagent (Invitrogen). The efficiency of ANXA2 expression knockdown was confirmed by western blotting and quantitative real-time RT-PCR assays.

### Progeny virus production measurements

We used the TCID_50_ assay to evaluate progeny virus production. Briefly, MDCK cells were prepared as a confluent culture in a 96-well plate 1 day prior to viral titration. After washing with PBS, the cells were incubated with serum-free Minimum Essential Medium (MEM) [46] and 1:10 serial dilutions of the cell supernatant containing the progeny viruses were adsorbed onto the plates in quadruplicate. After absorption for 1 h at 37 °C, the cells were washed twice and then incubated for an additional 48 h in MEM. The viral titers in the supernatants were measured by hemagglutination and the average value of three experiments was determined.

### Qrt-Pcr

Using TRIzol Reagent (Invitrogen) to extract total RNA from A549 cells, we next used a LightCycler480 (Roche, Basel, Switzerland) in conjunction with One Step SYBR® PrimeScript™ RT-PCR Kit II (Takara, Kyoto, Japan) to analyze viral gene expression. In this experiment, the housekeeping gene glyceraldehyde-3-phosphate dehydrogenase (GAPDH) was chosen as an internal control. The primer sequences for this experiment are shown in Table 2 **.**

### Statistical analysis

Viral titers and genome copy numbers are expressed as the mean ± standard error. GraphPad Prism 5 was used for Windows software (GraphPad Software, Inc.) to perform a Student’s t-test for analysis of statistical significance.

## Additional file

Additional file 1: Figure S1. NS1 protein of GD1322 distributed mainly in cytoplasm of A549 cells. (a) A549 cells were transfected with Flag-NS1, and fixed at 3, 6, 12, 24, 48 h post-transfected. The cells were immunostained for FLAG-NS1 by anti-Flag antibody (green) and for nucleus by DAPI (blue). (b) A549 cells were infected with GD1322, and fixed at 3, 6, 12, 24, 48 h post-infected. The cells were immunostained for NS1 proteins by D7 (green) and for nucleus by DAPI (blue). (TIFF 109022 kb)

## Abbreviations

AIV: Avian influenza virus
ANXA2: Annexin A2
co-IP: Coimmunoprecipitation
ED: Effector domain
IP: Immunoprecipitation
LTQ-MS: Linear ion trap Fourier transform ion cyclotron resonance-mass spectrometry
MOI: Multiplicity of infection
NS1: Non-structural protein 1
qRT-PCR: Quantitative Real-time RT-PCR
RBD: RNA-binding domain
TCID_50_: 50% tissue culture infectious dose
